# Effects of social disruption in elephants persist decades after culling

**DOI:** 10.1186/1742-9994-10-62

**Published:** 2013-10-23

**Authors:** Graeme Shannon, Rob Slotow, Sarah M Durant, Katito N Sayialel, Joyce Poole, Cynthia Moss, Karen McComb

**Affiliations:** 1 Mammal Vocal Communication & Cognition Research, School of Psychology, University of Sussex, Brighton BN1 9QH, UK; 2 Institute of Zoology, Zoological Society of London, Regents Park, London NW1 4RY, UK; 3 Amboseli Elephant Research Project, Amboseli Trust for Elephants, PO Box 15135, Langata, Nairobi 00509, Kenya; 4 Amarula Elephant Research Programme, School of Biological and Conservation Sciences, University of Kwazulu-Natal, Westville Campus, Pvt. Bag 54001, Durban 4000, South Africa; 5 ElephantVoices, Buskhelinga 3, Sandefjord 3236, Norway

**Keywords:** Social behaviour, Human disturbance, Anthropogenic disruption, Cognitive abilities, Playback experiment, Large-brained mammals, Social organisation, *Loxodonta africana*, Fission-fusion society, Vocal communication, Matriarch

## Abstract

**Background:**

Multi-level fission-fusion societies, characteristic of a number of large brained mammal species including some primates, cetaceans and elephants, are among the most complex and cognitively demanding animal social systems. Many free-ranging populations of these highly social mammals already face severe human disturbance, which is set to accelerate with projected anthropogenic environmental change. Despite this, our understanding of how such disruption affects core aspects of social functioning is still very limited.

**Results:**

We now use novel playback experiments to assess decision-making abilities integral to operating successfully within complex societies, and provide the first systematic evidence that fundamental social skills may be significantly impaired by anthropogenic disruption. African elephants *(Loxodonta africana)* that had experienced separation from family members and translocation during culling operations decades previously performed poorly on systematic tests of their social knowledge, failing to distinguish between callers on the basis of social familiarity. Moreover, elephants from the disrupted population showed no evidence of discriminating between callers when age-related cues simulated individuals on an increasing scale of social dominance, in sharp contrast to the undisturbed population where this core social ability was well developed.

**Conclusions:**

Key decision-making abilities that are fundamental to living in complex societies could be significantly altered in the long-term through exposure to severely disruptive events (e.g. culling and translocation). There is an assumption that wildlife responds to increasing pressure from human societies only in terms of demography, however our study demonstrates that the effects may be considerably more pervasive. These findings highlight the potential long-term negative consequences of acute social disruption in cognitively advanced species that live in close-knit kin-based societies, and alter our perspective on the health and functioning of populations that have been subjected to anthropogenic disturbance.

## Background

While we know that sociality evolves when the net benefits of close (often kin-based) associations with conspecifics outweigh the costs, there is still a lack of detailed information on how sociality translates into fitness consequences and the role of normative social structure in mediating these effects [1,2]. Nowhere is this issue more pertinent than in cognitively advanced social mammals such as some non-human primates, cetaceans and elephants which live in complex social systems where intricate social relationships develop over long lifespans and may involve cultural transmission of knowledge between generations [3-5]. Moreover, many free-ranging populations of these highly social mammals currently face extreme disturbance through human activities [6-8] that impacts directly on social structure, yet a proper understanding of how this “anthropogenic disruption” might affect core aspects of social functioning is lacking. Recent studies have started to highlight the significant long-term effects of disruptive events on physiological stress levels and broad behavioural patterns [9-12], but we still know very little of how fundamental skills of communication and cognitive abilities that are at the basis of such societies might be affected.

Anthropogenic disturbance of free-ranging populations can occur through processes such as illegal and legal hunting/culling, translocation and habitat fragmentation [7-9,13]. All of these are likely to be exacerbated further by increasing pressures on natural resources and climate change [14] and in extreme cases such impacts may result in significant loss of individuals. Disrupted populations typically experience two specific effects that are likely to impact on their social functioning - initial trauma that may accompany the disruptive event (which can involve survivors observing the killing of individuals around them) and the subsequent loss of opportunities for interacting with older group members that could act as appropriate role models or repositories of knowledge [3-5,15].

With regard to the first of these impacts, it is now becoming clear that, in animals as well as humans, social trauma experienced early in life may have very significant effects on physiological development and adult behaviour patterns [16-18]. For instance, in highly social and cognitively advanced species such as primates and elephants, where neurological development is strongly mediated by exposure to complex social information, a severely disruptive event can result in the expression of one or more non-normative behaviours during later life, including persistent fear, hyper-aggression and infant abandonment [19,20]. Dramatic consequences of social disruption have been documented in two protected areas in South Africa, where orphaned male elephants exhibited abnormal hyper-aggressive behaviour that resulted in the killing of 107 rhinoceroses over a period of 10-years [19,21,22]. Crucially, such traumatic events are also predicted to have more subtle effects on learning, in particular interfering with abilities to gauge appropriate responses to social and environmental stimuli [16-18].

The second major impact, namely a loss of opportunities for exposure to appropriate older role models, is likely to accompany any direct effects of social disruption on knowledge acquisition and decision-making. This is particularly relevant in long-lived and cognitively advanced species where older individuals play a key leadership role and co-ordinate decision-making in the context of social and ecological threats [3-5]. Where these experienced individuals are absent, younger group members may be presented with fewer opportunities to learn the most appropriate response in dangerous situations [3,4,23,24]. In addition, any abnormal behavioural patterns that have arisen from socially disruptive events have the potential to be passed between the generations and may persist in the long term.

By applying our previously successful playback techniques in two contrasting populations of African elephants we were able to assess directly effects of disruption on decision-making abilities integral to operating successfully within complex societies [3,4]. Our natural study population in Amboseli National Park, Kenya is relatively undisturbed in comparison with the population in Pilanesberg National Park, South Africa that was founded from young orphaned elephants introduced during the early 1980s and 1990s, following management culls of adult and older juvenile animals in the Kruger National Park [21,22,25]. These actions resulted in the young elephants being exposed to a significant traumatic event (the selective killing of all of their older family members followed by translocation to an unfamiliar environment), as well as the severe long-term damage to the core social unit - the family group - in this highly social species [16,19]. If social disruption impacts decision-making processes central to social functioning, we would predict deficits in abilities of the Pilanesberg elephants to respond appropriately to social threat.

### Playback experiments

Family units in both populations were presented with two complementary experimental paradigms involving standardised playbacks of female contact calls broadcast from a fieldwork vehicle located 100 m from the subjects (detailed in Methods). In the first experiment, we compared social knowledge directly in the two populations on the basis of subjects’ reactions to callers from three distinct social categories (high and low association index callers within the same population, constituting familiar versus unfamiliar associates, and alien callers from a separate population - Pilanesberg elephants in the case of Amboseli and vice versa: see Methods). The second experiment contrasted the responses of family groups in both populations to callers where age-related acoustic cues in re-synthesised calls simulated unknown individuals on an increasing scale of social dominance. Female elephants live in fission-fusion populations where social hierarchy is primarily based upon age, with older and larger individuals being more socially dominant than younger females, both within their respective groups [26] and during inter-group encounters [27,28]. The acoustic characteristics of five caller exemplars from each population (N = 10) were each systematically re-synthesised to simulate five different age classes of callers (15, 25, 35, 45 and 55 years), producing a set of 50 calls in total [see Methods & Additional file 1: Supplementary experimental procedures, and Additional file 2: Figure S1 & Table S1]. Amboseli elephants were only played caller exemplars from Pilanesberg (unknown individuals) and vice versa.

Four key behaviours (bunching, bunching intensity, prolonged listening and investigative smelling – see Methods for definitions) were used to test the responses of the elephant groups during the playback experiments. The reactions of all individuals within the family were recorded on video and systematically coded after the playback for analysis using generalised linear mixed models (GLMMs) in the R statistical program (Methods); results were confirmed with blind double coding by two independent observers (Methods). If subjects were able to discriminate effectively between callers in playbacks, we predicted that they should remain relatively relaxed when played calls that conveyed low levels of social threat - familiar or young individuals - and bunch into defensive formation and show heightened attentiveness when played calls representing high levels of social threat - unfamiliar or older individuals [3,4]. The ability to make these important distinctions should allow individual matriarchs to direct the overall group response most appropriately, and with the lowest cost and risk in relation to the specific threat at hand.

## Results

**Figure 1:**
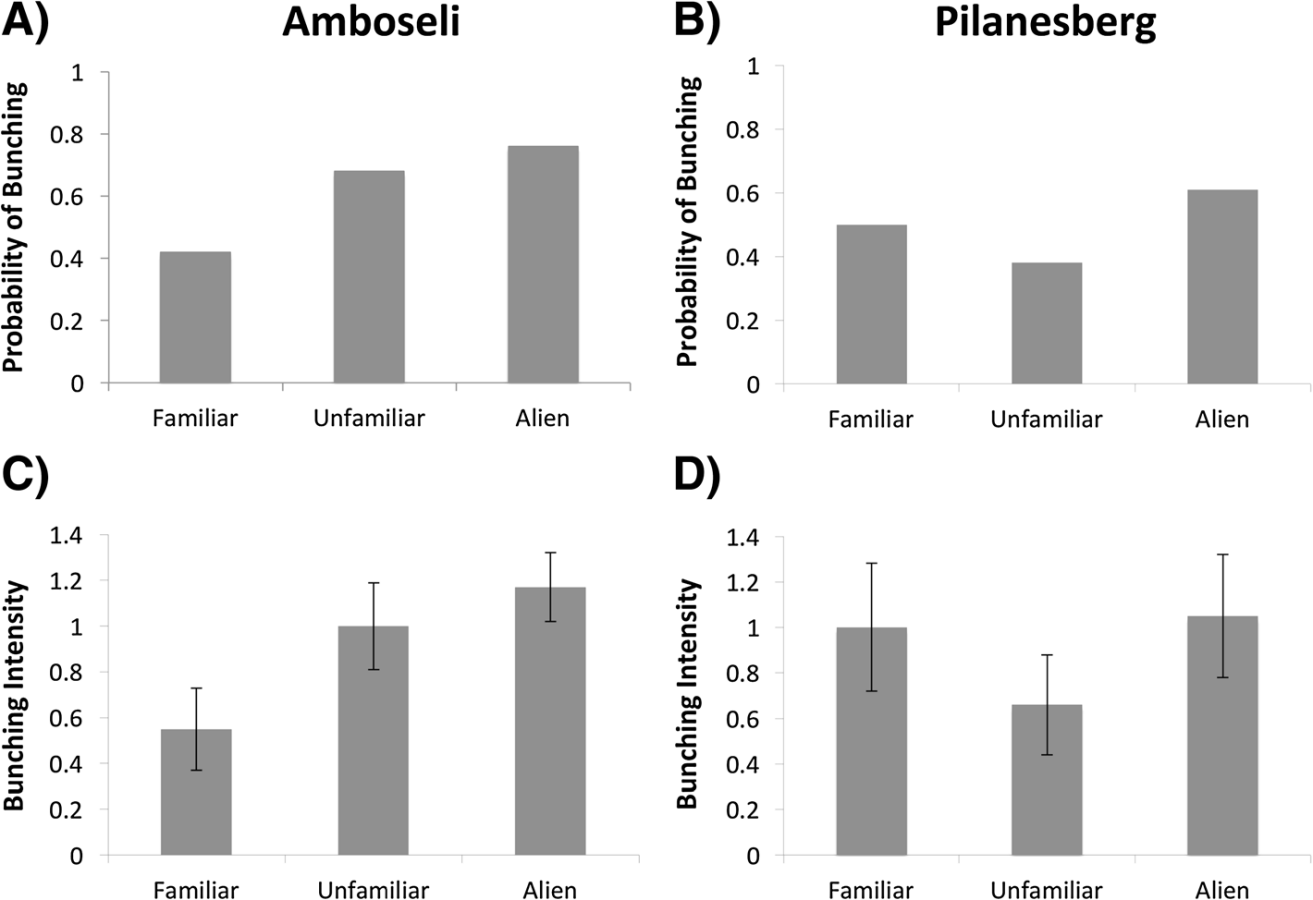
**Defensive bunching of elephant family groups in Amboseli (A & C) and Pilanesberg (B & D) to playbacks of contact calls from different social categories.** Behavioural responses were measured as probability of bunching **(A & B)** and mean (± s.e.m) bunching intensity **(C & D)**.

The first series of experiments demonstrated that elephants in the undisturbed Amboseli population distinguish between callers on the basis of their social category, focusing their defensive bunching on alien callers (GLMM analysis: Table 1A & Figure 1A). Our bunching intensity (Figure 1C), and prolonged listening measures also showed corresponding increases in response to alien callers, but in these cases the simpler null models were selected using Akaike’s information criterion adjusted for small sample sizes [AICc: see Additional file 3: Table S2A], indicating that this was a relatively weak response. By contrast, in Pilanesberg there was no evidence that any of the behavioural response variables significantly differed according to the social familiarity of the caller, and null models provided the best fit for the data in all cases (Table 1A; Figure 1B & D; Additional file 3: Table S2B). These results suggested poor abilities for social contextualisation among the Pilanesberg elephants [see also Additional file 4: Supplementary results].

**Table 1:** Results of GLMMs investigating the behavioural responses of elephant family groups to playbacks of contact calls that varied in social affiliation (experiment 1) and social dominance (experiment 2)

**A) Experiment 1:**		**Amboseli National Park**				**Pilanesberg National Park**			
**Dependent variable**	**Parameter**	**Estimate**	**s.e.**	**Z-value**	***p*-value**	**Estimate**	**s.e.**	**Z-value**	***p*-value**
Defensive bunching	Alien vs. familiar	1.476	0.579	2.548	0.01	0.471	0.703	0.670	0.50
	Unfamiliar vs. familiar	1.092	0.678	1.610	0.11	−0.525	0.682	−0.770	0.44
Bunching intensity	Alien vs. familiar	0.620	0.319	1.942	0.05	−0.042	0.341	−0.123	0.90
	Unfamiliar vs. familiar	0.406	0.373	1.088	0.28	−0.394	0.374	−1.053	0.29
Prolonged listening	Alien vs. familiar	1.322	0.635	2.080	0.04	−0.428	0.711	−0.602	0.55
	Unfamiliar vs. familiar	0.783	0.737	1.062	0.29	0.080	0.684	0.118	0.91
Investigative smelling	Alien vs. familiar	0.062	0.557	0.111	0.91	−0.868	0.727	−1.194	0.23
	Unfamiliar vs. familiar	0.506	0.683	0.740	0.46	−0.750	0.706	−1.062	0.29
**B) Experiment 2:**		**Amboseli National Park**				**Pilanesberg National Park**			
**Dependent variable**	**Parameter**	**Estimate**	**s.e.**	**Z-value**	***p*-value**	**Estimate**	**s.e.**	**Z-value**	***p*-value**
Defensive bunching	Age of caller	0.066	0.019	3.444	<0.001	0.0002	0.021	0.011	0.99
Bunching intensity	Age of caller	0.023	0.008	3.026	0.002	0.002	0.009	0.238	0.81
Prolonged listening	Age of caller	0.037	0.018	2.073	0.04	0.017	0.020	0.827	0.41
Investigative smelling	Age of caller	0.040	0.017	2.390	0.02	−0.032	0.021	−1.537	0.12

For experiment 1, the social affiliation parameter was categorical and the model generated results for the alien and unfamiliar playbacks using the familiar category as a reference. See also Additional file 3: Table S2 & Table S3.

However, the possibility remained that the contrasting pattern of responses described above could also be driven by differences in social attitudes between the populations. In particular, lack of opportunity to form bonds with kin when the Pilanesberg population was founded may conceivably have led to greater acceptance of unknown individuals [11,29]. Crucially therefore, our second series of experiments systematically tested for a core social skill that has direct functional relevance in both populations - the ability to discriminate between unknown callers on the basis of their social dominance [26-28]. Responding appropriately to more dominant individuals within the social hierarchy, and thus avoiding escalated interactions, is fundamental to emerging as successful within complex fission-fusion societies where individuals may come into contact with hundreds of others in the population as they move and feed [3,26-28]. Re-synthesis allowed us to manipulate fundamental (F0) and formant frequencies in the calls independently, while leaving other acoustic parameters unchanged, thereby creating standardised stimuli that were representative of callers of the five different ages (see Methods & Additional file 1: Supplementary experimental procedures and Additional file 2: Figure S1 & Table S1).

**Figure 2:**
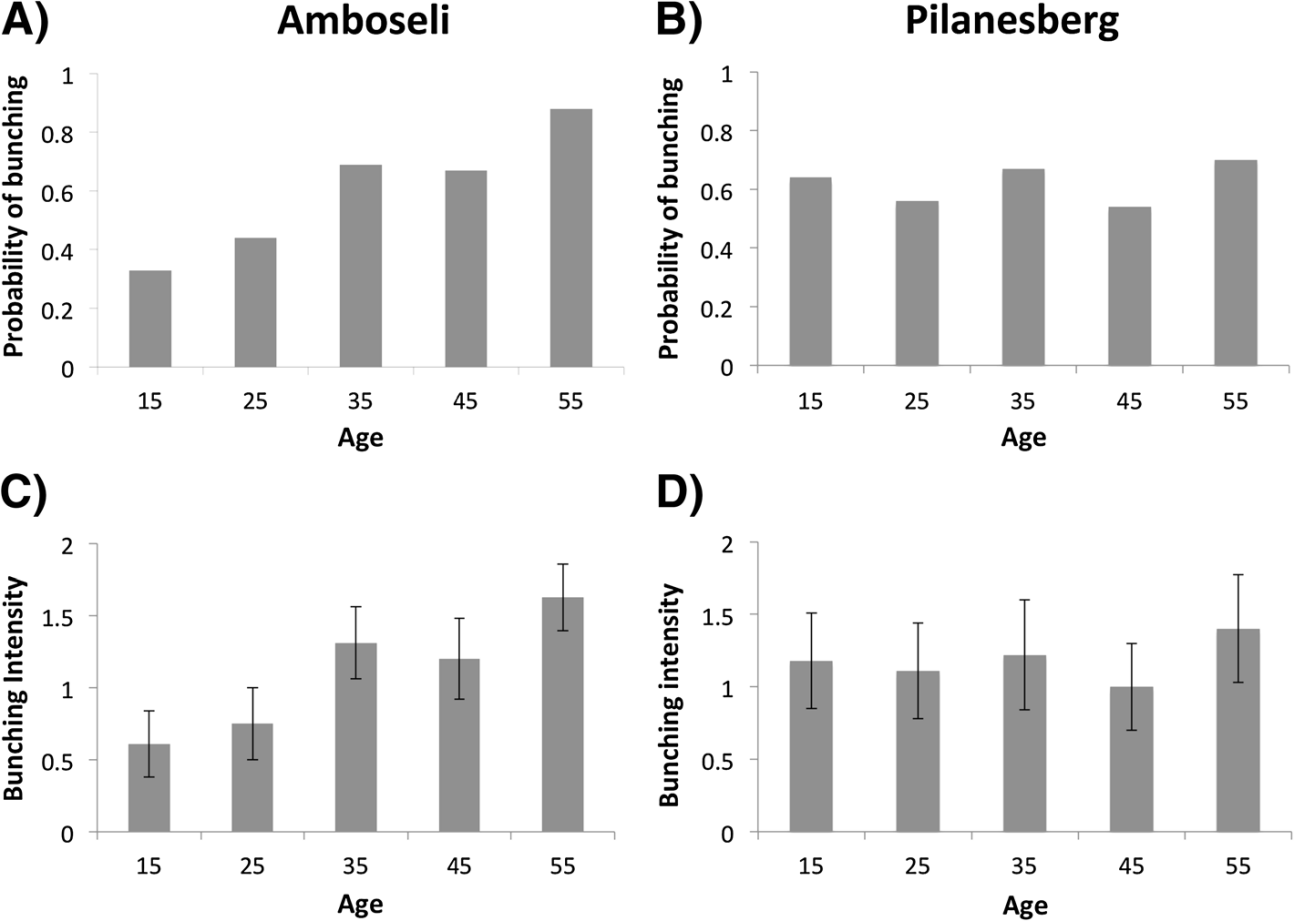
**Defensive bunching of elephant family groups in Amboseli (A & C) and Pilanesberg (B & D) to playbacks of re-synthesised contact calls simulating 5 different levels of social dominance on the basis of distinct age/size classes (see also Additional file****2****: Table S1 & Figure S1).** Behavioural responses were measured as the probability of bunching **(A & B)** and mean (± s.e.m) bunching intensity **(C & D)**.

In this main set of experiments our results clearly demonstrated that, while the Amboseli elephants discriminated between callers simulating different age classes and were most defensive to the oldest callers representing more socially dominant individuals (Table 1B; Figure 2A & C, Additional file 3: Table S3A), there were no such differences in discrimination abilities evident in the Pilanesberg population (Table 1B; Figure 2B & D, Additional file 3: Table S3B). In particular, there were marked contrasts in defensive bunching and bunching intensity in relation to age of caller in Amboseli, with the oldest callers (simulating more dominant individuals) eliciting more frequent and stronger defensive bunching reactions (Table 1B; Figure 2A & C). These results are also borne out in a direct comparison of the populations that revealed a significant difference in the sensitivity of the defensive bunching response of Amboseli elephants to the age of caller in our playbacks compared with subjects in Pilanesberg (GLMM: population × age of caller: Estimate = −0.066, Standard Error = 0.028, Z value = −2.333, *P* = 0.02). Furthermore, prolonged listening and investigative smelling reactions, both indicating attempts to gather additional information on the caller, increased significantly with caller age in Amboseli, as would be predicted if older callers were recognised as representing a greater threat. However, there was no evidence of an ability to make these same key distinctions in the Pilanesberg elephants (Table 1B).

It is important to note that while the lower maximum age of matriarchs in Pilanesberg (age range: 24–47 versus 23–70 in Amboseli) may have contributed to the poor social discrimination abilities evident here [3,4], it does not appear to have driven the results. In the basic social discrimination tests used in the current study there were no significant interactions between matriarch age and either social relationship with caller (experiment 1), or age of caller (experiment 2), in the best models for either of our study populations (see Additional file 3: Table S2 & Table S3). Moreover, when the oldest matriarchs (48 years and over) were removed from the Amboseli dataset for our main analyses, the results remained statistically significant [see Additional file 4: Supplementary results].

## Discussion

The ability to maintain important social relationships is believed to have direct fitness benefits for individuals, allowing them to maximise survival and reproductive success in constantly changing socio-ecological environments [1,2,30]. This is particularly apparent in large-brained, social species where information is accumulated over long life spans [1,3-5,27,31]. However, extremely disruptive events, including culling, poaching and translocation to new areas or capture for captivity can ultimately lead to serious disruption of the intricate social networks that underpin social structure in these species, with severe impacts on each individual’s close social bonds and opportunities for learning from older group members [9,11,16,19]. Furthermore, such disruption appears capable of driving aberrant behaviours in social animals that are akin to the post-traumatic stress disorder experienced by humans following extremely traumatic events [16,19]. While elephants in the wild can appear to exhibit short-term resilience following social disruption, apparently forming stable and reproductively active family groups (but see 9), the results presented here suggest that important decision-making abilities that are likely to impact on fundamental aspects of the elephant’s complex social behaviour may be significantly altered in the long-term.

Our work provides an unusual opportunity to examine directly links between social structure and inherent social skills that are at the basis of individual and group-level interactions in cognitively advanced mammals [1,2]. Cognition encompasses the mechanisms by which animals acquire, process, store and act on information from the environment, including perception, learning, memory and decision-making [32]. Responses in our two playback experiments suggest that functionally important decision-making abilities may be significantly altered by disruption of the natural structure of kin-based social relationships. Contrasting patterns of responses to socially unfamiliar elephants in our initial tests of social knowledge could conceivably be driven by differences in social attitudes, if lack of opportunities to bond with kin in the original Pilanesberg population resulted in greater acceptance of unknown individuals [11,29]. However, it is important to note that the Pilanesberg elephants did not show lower levels of defensive bunching overall - instead they simply failed to focus their defensive bunching on the most socially threatening individuals. Moreover, our main series of experiments subsequently tested for a social skill with direct functional relevance in both populations, the ability to assess age-related social dominance [26-28]. Here again, Pilanesberg elephants were apparently unable to distinguish between the level of social threat presented by older versus younger callers.

Previous studies have documented that a single traumatic event is sufficient to impact the neurological development of the mammalian brain [17,18,33,34], and the large hippocampus of the African elephant, which mediates social memory, is thought to be particularly susceptible during growth to adolescence [19]. The relative importance that such neurological changes might have in generating impaired decision-making versus the consequences of lack of exposure to older more experienced group members in the years following the traumatic event is hard to assess, but both may be important in driving our results. Exposure to older more experienced individuals has been shown to facilitate the development of functionally important skills in a range of mammals see [23,24] for reviews, and non-human primates deprived of appropriate role models acquire a smaller set of learned skills [23,35]. Although social learning has not been definitively demonstrated in wild African elephants, there is evidence that knowledge transfer does occur between experienced and naïve individuals [36] in common with many other large brained, socially complex species [23,24,37]. Further studies are now required to partition out these potential effects, and to assess their generality across populations that have experienced differing levels of disturbance.

Understanding the impacts of disrupting social bonds can both provide crucial insights into processes central to social evolution and also throw light on the functioning of advanced mammal societies that have been radically impacted by human disturbance. Our findings suggest that the health and social functioning of wild populations of long-lived and highly social species could be significantly impacted in the long-term by elevated levels of anthropogenic disturbance, which may compromise the ability of surviving individuals to respond appropriately to their conspecifics. Impairments to decision-making processes about threat may also contribute to the development of abnormally aggressive behaviour in response to other species, such as the killing of humans by female elephants in five populations established from translocated individuals that were the survivors of culls [38].

Although recent empirical evidence has highlighted the value of conserving functioning kin-based family groups, this remains an important issue that is often overlooked by wildlife practitioners in favour of population level management approaches that focus primarily on abundance [39]. In particular, while the recovery of populations from human-induced depletion is often assessed on the basis of numbers, it is now becoming clear that abnormal social structure may be a more persistent effect with very significant consequences [9,11,13,40,41]. These issues are currently very relevant, as translocation of mammal groups to new areas is becoming an increasingly common response in dealing with situations of animal-human conflict [29], whilst the escalation of poaching is having a dramatic effect on the structure of many populations [42]. Furthermore, in future years increasing demands on natural resources and ecosystem services from human societies is likely to intensify social disruption and conflict [14,43,44]. There is an assumption that wildlife responds to such pressures only in terms of demography, however our study demonstrates that cognitively advanced species such as elephants that live in complex societies may suffer more profound effects.

## Conclusions

By using playback experiments to systematically assess social discrimination skills in relation to developmental history, we provide the first direct evidence that abilities to process information on social identity and age-related dominance are severely compromised among African elephants that had experienced separation from family members and translocation decades previously. Long-lived species such as elephants, cetaceans and non-human-primates naturally exist in complex societies where behaviour and fitness is strongly affected by social relationships and exposure to older individuals is likely to influence knowledge acquisition by younger group members [1-5]. These critical facets of social living are often compromised in wild populations subjected to human disruption [9,11,40], and missing in the majority of captive environments [45]. Of particular concern, given the longevity of such species, is that the marked effects of these disruptions persist in the long-term.

## Methods

This work complies with the Association for the Study of Animal Behaviour/Animal Behaviour Society guidelines for the use of animals in research, and received approval from the Ethical Review Committee at the University of Sussex. We are grateful to the Kenyan Office of the President and to Kenya Wildlife Services for permission to conduct the research in Amboseli National Park, and to North West Parks and Tourism Board for permission to undertake this study in Pilanesberg National Park.

### Study populations

Fieldwork was conducted in Amboseli National Park, Kenya and Pilanesberg National Park, South Africa between February 2007 and November 2010. The elephant population in Amboseli numbered approximately 1500 individuals (including 58 family groups); in Pilanesberg there were approximately 200 individuals (including 16 family groups). The Amboseli Elephant Research Project has long-term demographic and behavioural data on the entire population, including detailed ages for all elephants born after 1971. The Pilanesberg population has been studied since 2000, with data available for the composition of each family group as well as ages for all of the adult females. Ages were estimated using criteria that are accepted as a standard in studies of African elephants [46].

### Sound recording and natural playback stimuli

Contact calls of adult female elephants (at least 11 years old) were used as playback stimuli for both experimental paradigms. These calls were recorded on digital audiotape using equipment specialized for low-frequency recording: a Sennheiser MKH 110 microphone linked to either a SonyTCD D10 DAT recorder (with DC modification) or a HHb PortaDAT PDR 1000 DAT recorder, through an Audio Engineering Ltd power supply (which incorporated a 5-Hz high-pass filter). With this equipment, the frequency response for recording was flat (±1 dB) down to at least 10 Hz. All contact calls used as stimuli were recorded in conditions of low air turbulence, at a distances of 30 m or less from particular known individual females, often calling in situations when they were separated from the rest of the group; calls were only included if the identity of the caller was completely unambiguous (see also [47,48]). The playback system used custom-built loudspeakers designed and constructed by Aylestone Ltd, Cambridge, UK and Bowers & Wilkins, Steyning, UK. The Aylestone system was composed of a custom-built sixth-order bass box loudspeaker with two sound ports linked to either a Kenwood KAC PS 400 M, Kenwood KAC923 or Kicker Impulse 1252 xi power amplifier and a HHb PortaDAT PDR 1000 DAT or Sony TCD D10 recorder (with DC modification), while the Bowser & Wilkins loudspeaker was powered by Alpine PDX-1.1000 and MRP-T222 amplifiers, linked to a Tascam HD-P2 digital audio recorder. Both playback systems had a lower frequency limit of 10 Hz and a response that is flat ±4 dB from approximately 15 Hz.

### Social categories in experiment 1

Prior to the playback experiments being carried out, individual family groups were assigned a contact call for each social category of caller (familiar, unfamiliar and alien), based on the observed level of association [see Additional file 1: Supplementary experimental procedures]. Callers from outside the population were categorised as alien as these individuals were unknown to the target family, while the callers from within each population were ranked from highest level of affiliation to the lowest using the association indices. The mean association index value was then calculated across these playbacks and used as a cut-off to categorise familiar (≥ mean level of association) and unfamiliar (< mean level of association) playback presentations for analysis.

### Re-synthesis of contact calls for experiment 2

Five individual contact calls were selected from each study population for re-synthesis, providing ten exemplars. Each of these exemplars was then re-synthesised with respect to age-related acoustic cues (fundamental frequency and formant frequencies) to produce five distinct contact calls per exemplar, simulating each female caller at 15, 25, 35, 45 & 55 years of age [see Additional file 1: Supplementary experimental procedures]. In this way, when presenting contact calls in playbacks, we controlled for individually distinctive acoustic characteristics of callers while systematically varying cues to their age and dominance. The ‘change gender’ function in PRAAT [49] was used to generate the appropriate new pitch median and the formant ratio shift (calculated by dividing the second formant frequency for the new re-synthesised age category by the frequency of the exemplar’s original second formant). This procedure was performed five times (number of age categories) for each of the ten exemplars. The spectrograms of the re-synthesised calls were viewed in PRAAT [49] to ensure that the pitch and formant frequencies had been adjusted correctly. Subjects were played stimuli from callers that are unknown to them (Amboseli elephants were exposed to stimuli from Pilanesberg and vice versa), so as to prevent any confounding effects resulting from recognition.

### Playback procedure

A total of 165 playbacks (experiment 1 n = 84, experiment 2 n = 81) were conducted in Amboseli and 109 (experiment 1 n = 57, experiment 2 n = 52) in Pilanesberg. An opportunistic approach was taken in selecting elephant family groups for inclusion in each experiment, which depended upon encountering the family within their home range in a relaxed behavioural state (e.g. foraging or resting). In Amboseli 39 families were selected for experiment 1 and 32 for experiment 2, while in Pilanesberg 14 families were selected for experiment 1 and 13 for experiment 2. Each family group was systematically played contact calls selected from the three categories of social affiliation (familiar, unfamiliar and alien), and the five re-synthesised age classes (15–55 years of age from the same exemplar) in randomised order. Each contact call was broadcast to the subjects from a fieldwork vehicle that was located 100 m from the periphery of the family group. The vehicle was positioned at right angles to the direct line of sight to the elephants, and the contact calls were played through the rear door from custom-built loudspeakers (see above). With this set-up the research vehicle, to which the elephants were habituated, acted as an effective visual barrier. Elephants have poor eyesight in comparison with their auditory and olfactory senses and typically respond to playbacks by listening and smelling in the direction of playback rather than trying to visually locate the caller [50]. Moreover, previous experiments in which the calling elephant was a relative revealed that the searching behaviour of subjects was consistent with them expecting the caller to be located in the area beyond the vehicle [47,48]. The peak sound pressure levels of the contact calls were standardised to 105 dB at 1 m (corresponding to the natural volume of a medium loud contact call). Sound pressure levels were measured with a CEL-414/3 sound level meter. A minimum period of seven days was left between playbacks to avoid habituation. Playbacks were not given to groups with calves of less than 1 month, as our previous work had indicated that the presence of such very young calves might result in abnormally high sensitivity to perceived threat [3].

The behavioural responses of the elephants to playback were observed through binoculars and recorded on a Canon XM2 video camera alongside live commentary. From video analysis we assessed five key behavioural measures that described the responses of the family group following playback (developed from [3,4]):

(1) Bunching: Defensive response to perceived threat by adult females and their young, which resulted in the diameter of the family group decreasing after the broadcast of a playback experiment (calculated in terms of elephant body lengths).

(2) Bunching intensity: The rate at which a defensive bunch of adult females and their young occurred. This measure classifies the overall level of threat response, scoring bunching intensity on a four-point scale as follows:

0 no bunching occurred

1 subtle reduction in diameter of the group, elephants remained relaxed and continue with pre-playback behaviours (> 3 min for bunch formation)

2 group formed a coordinated bunch, pre-playback behaviours such as feeding interrupted (1–3 min for bunch formation)

3 fast and sudden reduction in diameter of the group, elephants very alert (< 1 min for bunch formation)

(3) Prolonged listening: Adult female(s) continued to exhibit evidence of listening response for more than 3 minutes after playback, where ears are held in a stiff extended position, often with the head slightly raised.

(4) Investigative smelling: Adult female(s) engaged in either up trunk or down trunk smelling to gather olfactory information on the caller’s identity.

In the case of measures (3) and (4), each behaviour was scored as occurring if any adult female in the group engaged in that behaviour.

Two independent observers who did not have access to the live video commentary, and were blind to the playback sequence, second coded 25% of the video records comprising 68 videos (34 each); an overall agreement of 90% was achieved on the binary response variables (defensive bunching 96%, prolonged listening 90%, investigative smelling 85%) and the spearman’s ρ correlation on the scores for matriarch bunching intensity was 0.90 (*p* < 0.0001). It is important to note that the blind observers obtained this high level of agreement despite the fact that they were not able to score group behaviour that occasionally occurred off camera or some instances of smelling when a lowered trunk was obscured in the video (behaviours that were voiced on to the live commentary).

### Statistical analyses

The playback datasets were analysed separately for each elephant population using generalised linear mixed models (GLMMs) in the R statistical package [51]. The level of association with the caller (familiar, unfamiliar or alien) was used as the explanatory variable in the first experimental paradigm, while age of the call broadcast to the family group was used in the second. Four GLMM analyses were conducted, one for each of the key response behaviours (see above) that were selected as the dependent variables, while family group identity was entered as a random factor to account for repeated measures in the experimental design. Null models, which did not include any explanatory variables, were generated for each behavioural measure along with more complex models that investigated the additive and interactive effects of matriarch age and the number of adult females in the family group (variables used in our previous research as predictors of group-decision making 12 & 13 – see Additional file 4: Supplementary results). Model selection was performed using Akaike’s information criterion adjusted for small sample sizes (AICc) with lower AICc scores indicating better models; however, a more complex model with more degrees of freedom was only selected over a simpler model when the AICc differed by 2 or more [52].

## Competing interests

The authors declare that they have no competing interests.

## Authors’ contributions

KM, GS and RS designed the study and conceived the experiments. GS, KM and KS conducted the experiments. GS, KM and SD performed the statistical analysis and wrote the manuscript. JP and CM contributed essential data. All authors read, revised and approved the final version.

## Supplementary Material

Additional file 1 Supplementary experimental procedures. Click here for file

Additional file 2 **Age of caller and acoustic characteristics. Table S1.** Standard acoustic characteristics for setting the five different age categories of caller for the resynthesis experiment. **Figure S1.** Associated regression plots demonstrating the relationship between age of caller and two key acoustic parameters, A) fundamental frequency and B) the frequency of the second formant. Click here for file

Additional file 3 **Model selection using AICc. Table S2.** Model selection results of Generalised Linear Mixed Models (GLMMs) for the four key response behaviours of elephant matriarchs to playbacks of callers in different social categories. **Table S3.** Model selection results of Generalised Linear Mixed Models (GLMMs) for the four key response behaviours of elephant matriarchs to playbacks simulating callers of different levels of social dominance on the basis of distinct age/size classes. Click here for file

Additional file 4 Supplementary results. Click here for file
