# Supplementary material for: Effects of social disruption in elephants persist decades after culling
Source: Front Zool. 2013 Oct 23;10:62. doi: 10.1186/1742-9994-10-62 (PMC3874604; doi:10.1186/1742-9994-10-62)
Supplement: Additional file 1 — Supplementary experimental procedures. [file 1742-9994-10-62-S1.docx]

**Additional File 1: Supplementary Experimental Procedures**

**Analysis of acoustic cues to provide values for the re-synthesis of contact calls**

A total of 97 contact calls that had been recorded from 15 known adult female elephants (mean 6 ± 3 SD calls per female and age range 12 - 55 yrs) were used to examine age related cues in call characteristics. The calls were transferred from audio DAT tapes to digital WAV files. The PRAAT 5.2.21 DSP package [S1] was then used to carry out a low-pass filter (Pass Hann band settings 7 Hz to 10000 Hz with a smoothing factor of 1 Hz) for each of the calls before setting the new maximum amplitude at 95% of the original level using the ‘scale peak’ command. Calls were finally down-sampled to 0.551 kHz for analysis [following the procedure described in S2].

Source (fundamental frequency) and filter (formants) acoustic characteristics for each contact call [see S2] were extracted using commands in PRAAT [S1]. Firstly, individual pitch values were sampled across the call using an autocorrelation (To pitch (cc) command) algorithm that produced time-varying numerical representations of the fundamental frequency. The time step in the analysis was set at 0.1 s. These data points were exported as a text file to Microsoft Excel and the mean fundamental frequency calculated for each individual call. The formant frequencies were determined by viewing an overall spectrum of the call using the ‘To Spectrum’ command in PRAAT [S1]. An ‘LPC smoothing’ algorithm was then applied to yield values for the first five potential peaks in the 0–275.5 Hz range, which corresponded to individual formants. The second formant was the most consistent and defined across the entire range of calls and was therefore used as a reference for setting age related values. Regression analyses were employed in the final step of the procedure to determine the relationship between age of caller and the fundamental frequency (F_1, 13_ = 16.78, P < 0.01, r^2^ = 0.42) and second formant frequency (F_1, 13_ = 23.34, P < 0.001, r^2^ = 0.64) of the call. The regression line was used to calculate the appropriate fundamental frequency and second formant frequencies for female elephants aged between 10 and 55 yrs (Additional file 2, Table S1; Figure S1A and B). These values were then employed for preparation of our resynthesised playback stimuli.

**Calculating association indices between caller and family group**

Data on the ranging and association patterns of elephant family groups were obtained from regular daily monitoring at both study sites. These records include the location and composition of each family sighted. Using these data, association indices were calculated for the time period January 2002 to December 2007 as NAB/(NA + NB + NAB), where NAB = number of times families A and B are sighted in association in the same group; NA = number of times family A is sighted without B; and NB = number of times family B is sighted without A. The six-year period used for these analyses was determined by the duration of detailed daily monitoring in Pilanesberg, and ensured that both datasets spanned equal time frames as well as being sufficiently detailed to accurately determine levels of association.

**References**

S1. Boersma P, Weenink D, *Praat* (version 5.2.21 University of Amsterdam, The Netherlands) available for download www.praat.org (2011).

S2. McComb K, Reby D, Baker L, Moss C, Sayialel S (2003) Long-distance communication of acoustic cues to social identity in African elephants. Anim Behav 65: 317–329.
