# Supplementary material for: Effects of social disruption in elephants persist decades after culling
Source: Front Zool. 2013 Oct 23;10:62. doi: 10.1186/1742-9994-10-62 (PMC3874604; doi:10.1186/1742-9994-10-62)
Supplement: Additional file 2 — Age of caller and acoustic characteristics. Table S1. Standard acoustic characteristics for setting the five different age categories of caller for the resynthesis experiment. Figure S1. Associated regression plots demonstrating the relationship between age of caller and two key acoustic parameters, A) fundamental frequency and B) the frequency of the second formant. [file 1742-9994-10-62-S2.docx]

**Additional file 2: Age of caller and acoustic characteristics**

**Table S1.** Standard acoustic characteristics for setting the five different age categories of caller for the resynthesis experiment.

| Age of Caller | Pitch (Hz) | Second Formant (Hz) |
| --- | --- | --- |
| 15 | 17.5 | 119 |
| 25 | 16.0 | 112 |
| 35 | 15.5 | 106 |
| 45 | 15.0 | 102 |
| 55 | 14.0 | 99 |

**A**)

**B**)

**Figure S1.** Regression plots demonstrating the relationship between age of caller and two key acoustic parameters, A) fundamental frequency and B) the frequency of the second formant.
