# Supplementary material for: Effects of social disruption in elephants persist decades after culling
Source: Front Zool. 2013 Oct 23;10:62. doi: 10.1186/1742-9994-10-62 (PMC3874604; doi:10.1186/1742-9994-10-62)
Supplement: Additional file 3 — Model selection using AICc. Table S2. Model selection results of Generalised Linear Mixed Models (GLMMs) for the four key response behaviours of elephant matriarchs to playbacks of callers in different social categories. Table S3. Model selection results of Generalised Linear Mixed Models (GLMMs) for the four key response behaviours of elephant matriarchs to playbacks simulating callers of different levels of social dominance on the basis of distinct age/size classes. [file 1742-9994-10-62-S3.docx]

**Additional file 3: Model selection using AICc**

**Table S2.** Model selection results of Generalised Linear Mixed Models (GLMMs) for the four key response behaviours of elephant matriarchs to playbacks of callers in different social categories.

**A) AICc Values for Relationship caller – Amboseli**

|  | Null Model | Relationship Caller | Age Matriarch + Relationship Caller | Age Matriarch * Relationship Caller | No. Adult Females + Relationship Caller | No. Adult Females * Relationship Caller |
| --- | --- | --- | --- | --- | --- | --- |
| Defensive Bunch | 111.1 | 108.7  (*p*=0.01) | 110.7 | 114.3 | 110.8 | 112.6 |
| Bunching Intensity | 99.8 | 99.8  (*p*=0.05) | 101.9 | 105.3 | 98.8 | 100.7 |
| Prolong Listening | 117.5 | 116.8  (*p*=0.04) | 119.1 | 123.1 | 118.5 | 121.4 |
| Investigative Smelling | 117.5 | 121.5  (*p*=0.91) | 123.8 | 127.8 | 123.7 | 124.9 |

**B) AICc Values for Relationship caller - Pilanesberg**

|  | Null Model | Relationship Caller | Age Matriarch + Relationship Caller | Age Matriarch * Relationship Caller | No. Adult Females + Relationship Caller | No. Adult Females * Relationship Caller |
| --- | --- | --- | --- | --- | --- | --- |
| Defensive Bunch | 81.8 | 84.4  (*p*=0.50) | 86.4 | 85.6 | 83.6 | 88.6 |
| Bunching Intensity | 81.0 | 84.1  (*p*=0.90) | 86.2 | 84.6 | 85.7 | 90.5 |
| Prolong Listening | 81.1 | 85.1  (*p*=0.55) | 87.4 | 91.5 | 83.9 | 87.2 |
| Investigative Smelling | 80.0 | 82.9  (*p*=0.23) | 84.1 | 82.9 | 81.3 | 84.4 |

**Table S3.** Model selection results of Generalised Linear Mixed Models (GLMMs) for the four key response behaviours of elephant matriarchs to playbacks simulating callers of different levels of social dominance on the basis of distinct age/size classes

**A) AICc Values for Age of caller – Amboseli**

|  | Null Model | Age Caller | Age Matriarch + Age Caller | Age Matriarch * Age Caller | No. Adult Females + Age Caller | No. Adult Females * Age Caller |
| --- | --- | --- | --- | --- | --- | --- |
| Defensive Bunch | 113.5 | 102.6  (*p*=<0.001) | 101.1 | 103.2 | 103.2 | 98.9  (Interaction *p*=0.03) |
| Bunching Intensity | 107.7 | 100.4  (*p*=0.002) | 100.0 | 100.4 | 100.9 | 101.7 |
| Prolong Listening | 102.6 | 100.2  (*p*=0.04) | 93.8  (*p*=0.01) (*p*=0.02) | 95.0  (Interaction *p*=0.31) | 100.8 | 101.9 |
| Investigative Smelling | 115.8 | 111.9  (*p*=0.02) | 113.8 | 115.8 | 107.3  (*p*=0.02) (*p*=0.02) | 109.2 |

**B) AICc Values for Age of caller – Pilanesberg**

|  | Null Model | Age Caller | Age Matriarch + Age Caller | Age Matriarch * Age Caller | No. Adult Females + Age Caller | No. Adult Females * Age Caller |
| --- | --- | --- | --- | --- | --- | --- |
| Defensive Bunch | 72.7 | 75.0  (*p*=0.99) | 77.2 | 80.8 | 73.7 | 75.6 |
| Bunching Intensity | 69.7 | 71.9  (*p*=0.81) | 74.2 | 75.9 | 72.1 | 73.1 |
| Prolong Listening | 74.4 | 76.0  (*p*=0.41) | 77.1 | 79.6 | 78.3 | 80.1 |
| Investigative Smelling | 72.5 | 72.3  (*p*=0.12) | 70.4  (*p*=0.06) (*p*=0.14) | 72.5 | 74.6 | 74.4 |
