# Supplementary material for: Effects of social disruption in elephants persist decades after culling
Source: Front Zool. 2013 Oct 23;10:62. doi: 10.1186/1742-9994-10-62 (PMC3874604; doi:10.1186/1742-9994-10-62)
Supplement: Additional file 4 — Supplementary results. [file 1742-9994-10-62-S4.docx]

**Additional file 4: Supplementary Results**

**Detailed results of model selection and model fit**

The null models consistently provided the greatest level of support for GLMM analyses of the Pilanesberg datasets, with only investigative smelling and age of caller (experiment 2) providing a marginally better level of fit [Additional file 3, Tables S2B and S3B]. In Amboseli the simple models with a single explanatory variable (relationship or caller or age of caller) had AICc scores that were ≥2 points lower than the null models for defensive bunching in the first experimental paradigm and for all four behavioural measures (defensive bunching, bunching intensity, prolonged listening and investigative smelling) in the second experimental paradigm [Additional file 3, Tables S2A and S3A].

The introduction of matriarch age and number of adult females in the more complex models did not improve the model fit for any of the behavioural measures in Pilanesberg [Additional file 3, Tables S2B and S3B], or for the first experimental paradigm in Amboseli [Additional file 3, Table S2A]. However in experiment 2, three more complex additive and interactive models provided lower AICc scores when compared with the simple model [Additional file 3, Table S3A]. More specifically, groups with larger numbers of adult females were more likely to engage in investigative smelling and demonstrated a greater probability of defensive bunching to older callers compared with smaller groups, while older matriarchs demonstrated a greater overall probability of prolonged listening than younger matriarchs.

To confirm that our results were robust despite the inherent differences in the number of families in the two populations and the playback sample sizes, a random subset of data was drawn from both Amboseli datasets (matching those of Pilanesberg), and analysed using defensive bunching as the response variable - our key behavioural measure (Experiment 1: Amboseli n = 49, Pilanesberg n = 57; Experiment 2: Amboseli n = 51, Pilanesberg n =52). Twenty family groups from Amboseli were included in the age-matched dataset for experiment 1 and 30 were included in the age-matched dataset for experiment 2. The results of these analyses on the reduced datasets demonstrated that the relationship was still strongly significant for both experiments, with increased defensive bunching of Amboseli elephants to unknown alien callers in experiment 1 (Estimate =1.818, Standard Error = 0.767, Z value = 2.370, *P* value = 0.02) and with increasing age of the caller in experiment 2 (Estimate = 0.078, Standard Error = 0.026, Z value = 3.005, *P* value = 0.003). Furthermore, an age-matched analysis of the Amboseli dataset where only family groups with matriarchs ≤47 years of age were included (corresponding with Pilanesberg), demonstrated that matriarch age did not affect the ability of the Amboseli elephants to discriminate between callers on the basis of social dominance (Defensive bunching: Estimate = 0.066, Standard Error = 0.020, Z value = 3.368, *P* value = <0.001, Bunching intensity: Estimate = 0.029, Standard Error = 0.008, Z value = 3.466, *P* value = <0.001, Investigative smelling: Estimate =0.046, Standard Error = 0.018, Z value = 2.599, *P* value = 0.01, Prolonged listening: Estimate =0.031, Standard Error = 0.019, Z value = 1.641, *P* value = 0.1).
